# Identification of the efficacy and safety of apomorphine in the treatment of Parkinson’s disease via meta-analysis

**DOI:** 10.1186/s12883-025-04516-7

**Published:** 2025-11-27

**Authors:** Haidong Li, Liwen Zhang, Baona Jiang, Lu Jing

**Affiliations:** 1 Department of Neurology, The First Affiliated Hospital of Dalian Medical University, Dalian, China; 2 Science and Technology Department of Dalian Medical University, Dalian, China

**Keywords:** Parkinson's disease, Apomorphine, Meta-analysis

## Abstract

**Background:**

The majority of Parkinson’s disease (PD) patients experience motor fluctuations and dyskinesias as the disease progresses. Apomorphine has been used as an acute treatment for off episodes in PD in recent years. However, conclusions from clinical studies regarding the efficacy and safety of apomorphine have been inconsistent, particularly prior to the publication of larger pivotal trials.

**Objectives:**

To systematically evaluate the efficacy and safety of apomorphine in PD patients, a meta-analysis of randomized controlled trials (RCTs) and single-armed studies were performed.

**Methods:**

PubMed, Web of Science, Embase, and Cochrane databases were searched to identify all related studies. Outcomes of interest were changes in Parkinson’s Disease Rating Scale Part III (UPDRS-III) scores, ON and OFF time, and adverse events (AES).

**Results:**

In this meta-analysis, 20 clinical studies were included (*n* = 1262 patients): seventeen RCTs, and three single-armed studies. The pooled results from RCTs indicate that apomorphine treatment significantly improved patients’ motor function compared to placebo. Specifically, there was a mean difference (MD) of -12.28 (95% CI: [-17.98, -6.59]) in UPDRS-III scores. Patients treated with apomorphine experienced a reduced off time (MD = -10.70, 95% CI: [-20.33, -1.06]), indicating a shorter duration of the “off” state. Furthermore, the combined results indicated a conversion rate from off to on state in PD patients treated with apomorphine of 0.79 (95% CI 0.67–0.89). In terms of safety analyses from single-armed studies, apomorphine treatment was associated with several AES, including nausea, drowsiness, vomiting, dizziness, and excessive sweating. However, the findings suggest that the incidence of AES was relatively low during treatment with apomorphine.

**Conclusion:**

The meta-analysis results indicate that apomorphine effectively improves dyskinesia and motor fluctuations in patients with PD. Furthermore, the incidence of AES was relatively low during treatment with apomorphine.

## Highlights

• Our meta-analysis from RCTs and real-world observational studies confirmed that.

apomorphine effectively improves dyskinesia and motor fluctuations in patients with PD.

• In terms of safety analyses from single-armed studies, apomorphine treatment was associated with several AES, including nausea, drowsiness, vomiting, dizziness, and excessive sweating. However, the findings suggest that the incidence of AES was relatively low during treatment with apomorphine.

• Further trails are needed to assess the longer-term safety and efficacy of apomorphine as add-on treatment and as monotherapy.

## Introduction

 Parkinson’s disease (PD) is a progressive neurodegenerative disorder characterized by misfolded aggregates of alpha-synuclein and loss of dopaminergic neurons produced in the nigra of the brain [1]. PD is widely regarded as the second most common degenerative lesion of the central nervous system after Alzheimer’s disease [2]. The proportion of undiagnosed cases identified through screening increased from 18% in individuals aged 65–70 to 36% in individuals aged 80–85 [3]. Concurrently, the incidence, prevalence, and risk of mortality from PD are higher in men than in women, with a ratio of approximately 1.4:1 [3]. The main goal of pharmacological treatment of PD is to restore dopaminergic neurons and alleviate motor deficits. As a dopamine precursor, Levodopa is the first-line treatment option for enhancing motor function and prolonging survival in PD patients [4]. The key pathological feature of PD is the progressive degeneration of dopaminergic neurons in the substantia nigra pars compacta, leading to a sharp decline in striatal dopamine levels [5]. Concurrently, prolonged use of levodopa induces oxidative stress during the drug’s metabolic processes within the body [6]. Some hypotheses suggest that long-term levodopa exposure might contribute to neurodegenerative processes; however, the primary driver of disease progression is the underlying loss of dopaminergic neurons [7, 8]. These neurons synthesize and release dopamine into the striatum, a brain region critical for motor control [8, 9]. The progressive loss of dopaminergic neurons is a hallmark of PD. This leads to a severe depletion of striatal dopamine, which directly causes the characteristic motor symptoms of the disease. Furthermore, as the condition advances, patients often develop motor complications, including motor complications and dyskinesia [1, 7, 10]. Motor fluctuations are characterized by alternating between periods of good response to levodopa (‘on’ state) and periods of worsening PD symptoms (‘off’ state), which significantly impacting the performance of daily life in PD patients [11]. 

Currently, dopamine agonists are widely used for managing motor fluctuations in PD patients [12]. These drugs act by directly binding to dopamine D2 receptors, thereby enhancing dopamine D2 receptor activity. Apomorphine, a short-acting D1- and D2-like agonist, stands out due to its high lipophilicity, which allows it to rapidly penetrate the blood-brain barrier and be absorbed quickly by the brain [13, 14]. In recent years, various administration modes have emerged, including sublingual [15] and inhalation [16]. These routes significantly influence apomorphine’s efficacy and safety in PD treatment, affecting dyskinesia improvement, onset speed, and adverse event frequency [17]. Despite the well-established clinical efficacy of apomorphine subcutaneous infusion therapy in alleviating motor fluctuations in PD patients [18], most clinical trials have been open-label studies or controlled observations, leading to inconsistent conclusions regarding the efficacy of apomorphine therapy. A comprehensive meta-analysis validating apomorphine’s superiority over other therapies in relieving PD symptoms with a high level of evidence-based medicine is lacking. In a multicenter, double-blind, randomized, placebo-controlled trial (the TOLEDO study), Katzenschlager et al. enrolled 107 PD patients and demonstrated a significant reduction in patients’ “off periods” compared to placebo over a 12-week treatment period [19]. Additionally, patients in the apomorphine group experienced an average reduction in levodopa equivalent dose of nearly 500 mg, significantly higher than the reduction in the placebo group (*p* = 0.0014). This finding suggests that, in the context of optimized combination therapy, effective management of motor fluctuations with apomorphine may allow for a reduction in the dosage of other antiparkinsonian medications in some patients [19]. Subcutaneous administration of apomorphine is now well established for treating severe motor fluctuations and has been shown to steadily and consistently reduce the duration of the “off” state [14]. On average, patients in the apomorphine group experienced a reduction in daily “off” time of approximately 2 h compared to approximately 1 h in the placebo group, a statistically significant treatment difference (*p* < 0.05) [19]. For instance, while subcutaneous administration maintains stable plasma levels, effectively reducing the “off” state duration, it may be linked to higher adverse effects [18]. Conversely, sublingual or inhalation methods likely offer quicker onset, enhance the “on” state duration, and potentially carry fewer side effects [15, 16]. A comprehensive meta-analysis evaluating the efficacy and safety of apomorphine across different administration routes would enhance our understanding of PD therapeutic options and facilitate future clinical investigations. Such an analysis would provide detailed insights from placebo-controlled trials, offering evidence on apomorphine’s effectiveness in managing motor fluctuations and dyskinesias in PD patients. This evidence-based approach aims to guide more precise medication decisions in clinical practice, ultimately improving patient quality of life and treatment satisfaction.

## Materials and methods

### Search strategy

This Meta-analysis was registered in https://www.crd.york.ac.uk/PROSPERO/; Registration number: CRD42024529174. Up until April 2, 2024, we conducted searches across four international databases: PubMed, Web of Science, Embase, and Cochrane. Our search terms included (Parkinson’s disease OR Parkinsonism OR PD OR Parkinson’s disease’) AND (Apomorphine) in Title/abstract. Additionally, we manually searched references in clinical trial reports and reviewed articles to identify other pertinent studies.

### Selection criteria

Following a rigorous screening process conducted by two independent researchers, all titles and abstracts were evaluated against predefined inclusion and exclusion criteria. Any disputes during the screening process were resolved through discussion; unresolved issues were referred to a third researcher for adjudication. The inclusion criteria were as follows: (1) studies conducted in PD patients with motor fluctuations and dyskinesias; (2) use of apomorphine as the therapeutic intervention; (3) inclusion of a control group treated with placebo or levodopa; (4) assessment of motor fluctuations and dyskinesias as primary outcomes; (5) randomized controlled trial design or; retrospective or prospective observational studies or other interventional studies (6) participants aged 18 years or older. Exclusion criteria included: (1) studies involving animal experiments or reviews; (2) trials with inadequate or flawed data; and (3) patients lacking informed consent or ethical review approval.

### Data extraction

Two independent reviewers independently extracted the following data from included studies: first author, year of publication, study region, study design type (double-blind or open-label trial), sample size, age of experimental and control groups, details of intervention and route of administration, duration of illness, study duration, and key efficacy indicators.

### Assessed outcomes

The primary efficacy indicators included in the study were the Unified Parkinson’s Disease Rating Scale Part III (UPDRS-III), ON-TIME per day without troublesome dyskinesia, and the number and time taken for patients to transition from OFF-TIME to ON-TIME post-drug administration. Secondary endpoints comprised ratings for dyskinesia on any scale, UPDRS Parts II and PD Questionnaire 39 (PDQ-39), along with PD Questionnaire 8 (PDQ-8). Safety outcomes assessed the incidence of adverse events (AEs), such as nausea, drowsiness, vomiting, dizziness, and excessive sweating, as well as neuropsychiatric complications, movement disorders, and cutaneous reactions.

### Assessment of risk of bias

Two researchers assessed the risk of bias in randomized controlled trials using the Cochrane risk of bias tool. The tool evaluates bias across seven domains: (1) random sequence generation; (2) allocation concealment; (3) blinding of participants and personnel; (4) blinding of outcome assessment; (5) incomplete outcome data; (6) selective reporting; and (7) other sources of bias. Bias within each domain was classified as low risk, high risk, or unclear. In cases where the two researchers disagreed and consensus could not be reached through discussion, a third investigator was consulted for resolution.

Meta-analyses were performed when more than two studies had available data to assess outcomes. We used Review Manager version 5.4 and Stata version 18.0 to analyze the extracted data. Effect sizes and 95% confidence intervals (CIs) were calculated. For continuous data, effect sizes were calculated using mean and standard deviation (SD) and 95% CI, comparing the change in apomorphine versus control before versus after treatment. Single-arm analyses were performed to calculate ES and 95% CI for adverse effects. When significant heterogeneity existed, subgroup analyses were performed based on differences in the characteristics of the study population. Heterogeneity was tested based on I²; a random-effects model was chosen when I²>50% and a fixed-effects model was chosen when I²<50%. Publication bias was assessed using Egger’s test when more than ten studies were included. All statistical tests were two-sided, and a P-value of less than 0.05 was considered statistically significant. Also, by combining the results of Egger’s test with the visual presentation of funnel plots, we ensured a comprehensive consideration of publication bias.

## Result

### Literature selection

**Fig. 1:**
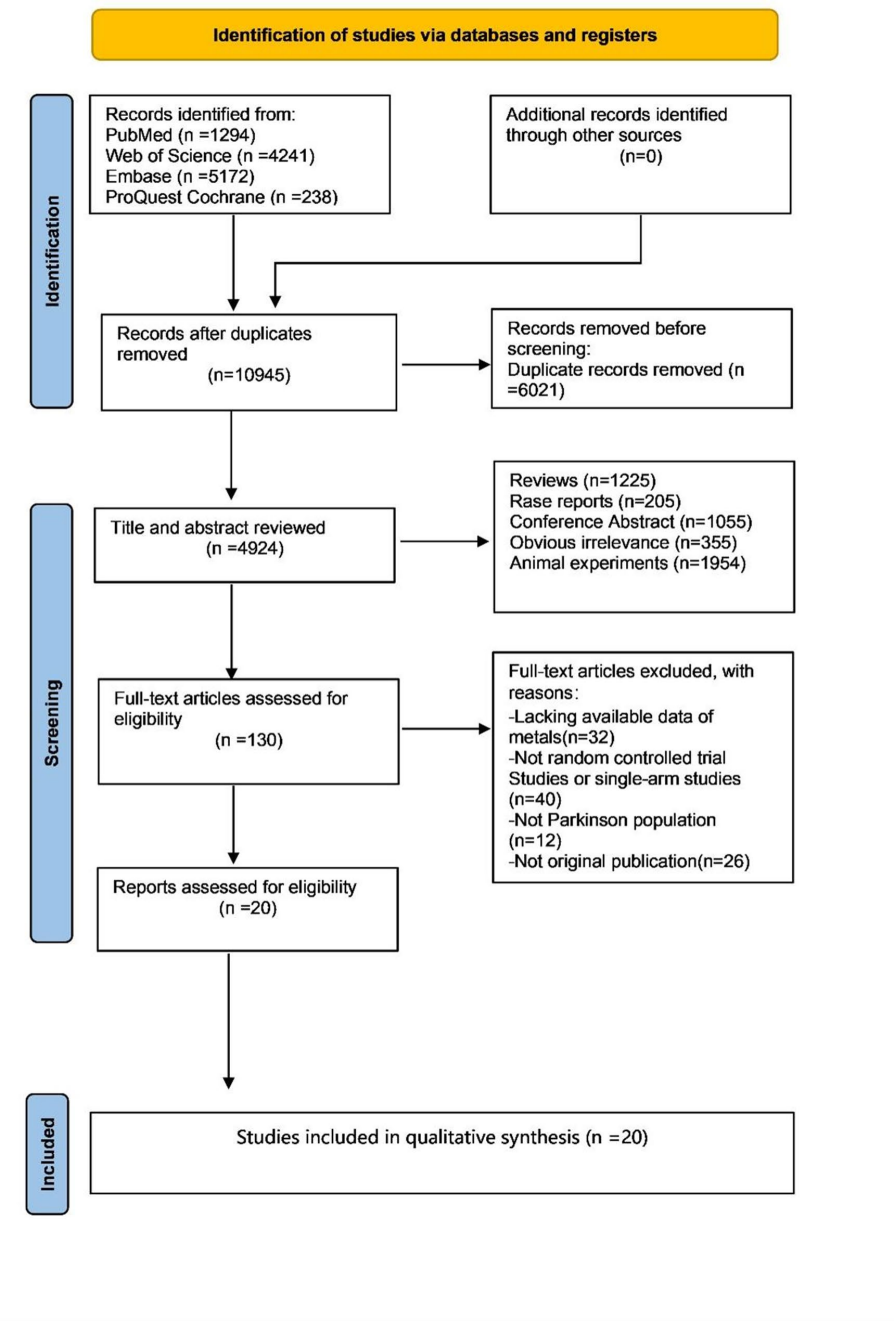
Flow diagram of study process

Figure 1 depicts the process of study selection. Initially, a total of 10,945 studies were identified from PubMed, Web of Science, Embase and Cochrane Library databases, and 4924 studies were identified after excluding duplicate studies. Of these, 4794 studies were excluded due to obvious irrelevance, animal studies, reviews, and conference abstract. After detailed assessment of remaining 130 full-text articles, 110 studies were further excluded since they did not meet the inclusion criteria, couldn’t extract original data, not apomorphine use in patients with PD. Ultimately, 20 studies (seventeen RCTs, and three single-armed studies) involving 1,262 PD patients were included in the meta-analysis (Fig. 1).

### Baseline characteristics of included literature

A total of 20 clinical studies [19–38] involving 1262 PD patients were included in this study. Detailed descriptions of included studies were summarized (Table 1). The effects of apomorphine PD patients were analyzed in terms of collecting seven clinical study outcomes, including UPDRS III score, daily Off time, off time change, number of people off to on state after dosing, off time after dosing, duration of on state after dosing, and incidence of AEs occurring in the treatment (nausea, vomiting, drowsiness, dizziness, sweating).

**Table 1:** Summary characteristics of included studies

First author (publication year) Study design and location	Intervention	Route of administration	Dosage of medication	Number of patients	Male/Female	age	PD duration	Primary results
K. A. Grosset 2013 [36] RCTs United Kingdom	Apomorphine	Inhale	0.2,0.5,0.8 mg	18	15/3	58	NA	1,7
	Placebo			6	5/1	56.3	NA	
Nobutaka Hattori 2014 [33] RCTs Japan	Placebo/apomorphine	Subcutaneous injection	1–5 mg	15	7/8	64.3 ± 10.9	12.5	1.,4,5,6,7
	Apomorphine/placebo			13	8/5	58.5 ± 9.9	12.2	
Regina Katzenschlager 2018 [19] RCTs Austria	Apomorphine	Subcutaneous injection	3–8 mg	53	34/19	63.6 ± 9.3	11.8 ± 5.6	2,3,7
	Placebo			53	32/21	63.0 ± 8.3	10.6 ± 4.3	
C. Warren Olanow2021 [27] RCTs United States	Placebo/apomorphine	Sublingual lozenges	10–60 mg	35	NA	63.9	8.4	1,7
Jennifer S. Hui2020 [31] RCTs United States	Apomorphine	Take sublingually	10–30 mg	109	NA	NA	NA	1,4,5,7
	Placebo			108	NA	NA	NA	
Fabrizio Stocchi2023 [23] RCTs Italy	Apomorphine	Sublingual lozenges	15–30 mg	112	78/34	64.4 ± 8.8	9.2 ± 4.2	1,7
	Apomorphine	Subcutaneous injection	2–6 mg					
C Warren Olanow2020 [28] RCTs United States	Apomorphine	Sublingual lozenges		54	37/17	62.9 ± 9.79	8.7 ± 4.25	1,4,5,7
	Placebo			55	31/24	62.5 ± 8.12	9.3 ± 3.84	
Fabrizio Stocchi2022 [24] RCTs Italy	Apomorphine	Sublingual lozenges	10–60 mg	48	30/18	64.9 ± 8.58	8.5 ± 4.36	7
	Crossover crowds			40	26/14	63.7 ± 8.68	8.3 ± 4.32	
Regina Katzenschlager2020 [30] Open labeling Austria	Apomorphine	Subcutaneous injection	0.1 mg/kg/h	84	NA	64.3 ± 8.2	10.9 ± 5.0±	3,7
Masahiro Nomoto2015 [29] RCTs Japan	Apomorphine	Subcutaneous injection	2–6 mg	10	4/6	57.7 ± 11.4	9.07 ± 4.58	1,5,6,7
	Placebo			6	1/5	58.8 ± 5.3	11.58 ± 5.05	
Robert A. Hauser, MD2016 [32] Open-label study United States	Apomorphine	Sublingual lozenges	10–30 mg	19	14/5	61.5 ± 8.7	NA	1,4,6,7
Eva Thijssen2022 [21] RCTs Netherlands	Apomorphine	inhale	2–4 mg	20	6/14	63.2 ± 11.1	NA	1,7
	Placebo			6	1/5	68.7 ± 4.6	NA	
Katherine A. Grosset2013 [35] RCTs United Kingdom	Apomorphine	inhale	1.5–4.5.5.5 mg	40	20/20	65.6 ± 7.7	12.2 ± 3.9	1,3,4,5,7
	Placebo			15	8/7	65.8 ± 5.7	11.5 ± 3.2	
Grosset KA2013 [34] RCTs United Kingdom	Apomorphine	inhale	1.5–4.0.5.0 mg	32	26/6	61.3		1,5,6,7
	Placebo			15	12/3	59.1		
Valérie Cochen De Cock2022 [38] RCTs France	Apomorphine	subcutaneous injection	5 mg/h	46	27/19	63.6 ± 9.2	9.8 ± 4.5	7
	Placebo							
Eva Thijssen, MSc2022 [22] RCTs Netherlands	Apomorphine	inhale	1 mg	38	16/22	40	NA	7
	Apomorphine	subcutaneous injection	2 mg		3/5	40		
	Placebo			10	8/2	40		1,7
Richard M. Trosch2008 [20] Open-label study United States	Apomorphine	subcutaneous injection	2–10 mg	51	31/20	66.1 ± 1.3	NA	
Richard B. Dewey2001 [37] RCTs United States	Apomorphine	subcutaneous injection	2–10 mg	20	12/8	66 ± 8.9	9.2 ± 4.9	1,4,5,7
	Placebo			9	8/1	62 ± 12	12.3 ± 6.3	
Ronald F. Pfeiffer2006 [25] RCTs United States	Apomorphine	subcutaneous injection	1.5–10.0 mg	35	25/10	64.8 ± 1.5	NA	1,7
	Placebo			27	20/7	66.5 ± 1.9		
Rajesh Pahwa2007 [26] RCTs United States	Placebo/Apomorphine	subcutaneous injection	2–10 mg	25	11/14	NA	55.6 ± 1.87	1,7
	Apomorphine/Placebo			26	16/10		54.8 ± 1.97	

Adverse events; *NA* Not available

*RCTs* Randomized controlled trials; Primary results: 1, UPDRS III score; 2, Off time (h per day); 3, Off time change/h; 4, Number of people changing from off to on; 5, Time from off to on; 6, Duration of on; 7

### Risk of bias

**Fig. 2:**
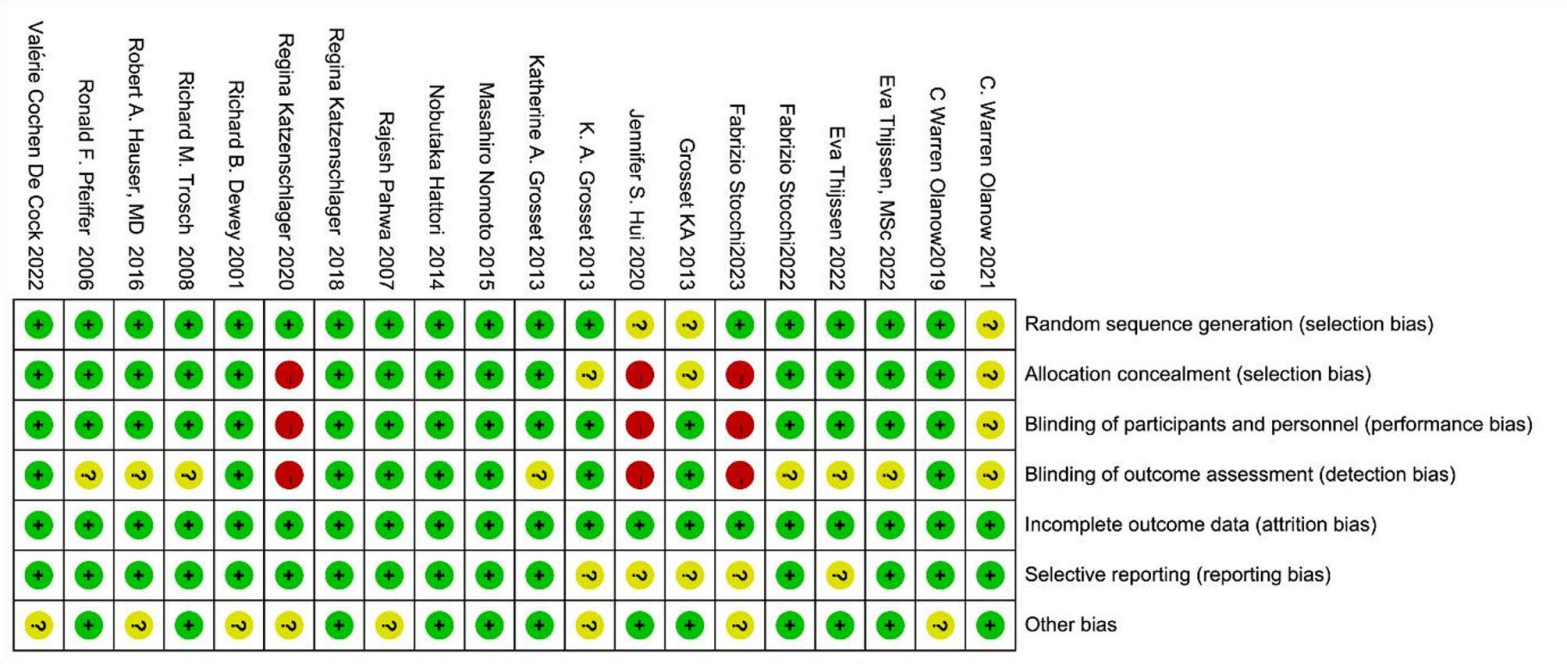
Risk of bias graph

Most trials were assessed as having a low risk of bias, particularly in RCTs where blinding of subjects and testers was effectively implemented. In contrast, open-label studies such as those by Jennifer S. Hui 2020, Regina Katzenschlager 2020, and Fabrizio Stocchi 2023 were rated as having a high risk of bias due to the inherent nature of their design, which posed risks of selection bias, performance bias, and detection bias, as illustrated in Fig. 2.

### Effect of apomorphine in reducing UPDRS III scores in patients with PD

**Fig. 3:**
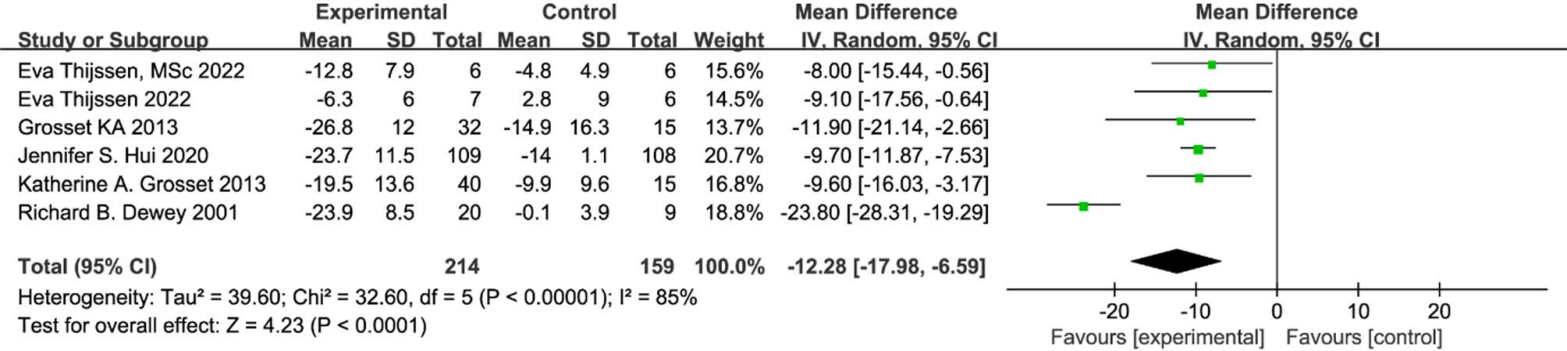
Meta-analysis of reducing UPDRS III scores in six RCTs

**Fig. 4:**
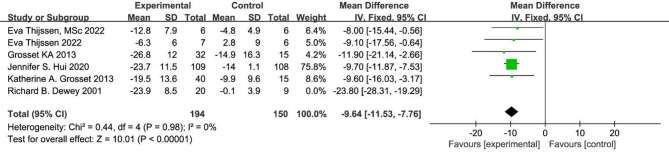
Meta-analysis of UPDRS part III score in five RCTs

Six studies [21, 22, 31, 34, 35, 37] were included in the meta-analysis. The overall effect sizes indicated a significant reduction in UPDRS III scores (MD = −12.28, 95% CI [−17.98, −6.59]) compared to placebo (Fig. 3). Furthermore, apomorphine exhibited statistically significant improvements in dyskinesia among PD patients (*p* < 0.0001).The initial six studies showed high heterogeneity (I² = 85%). Upon visual inspection and sensitivity analyses of effect sizes and confidence intervals for each study, it was noted that the study by Richard B. Dewey 2001 exhibited significantly different effect sizes compared to the other studies. After excluding the Richard B. Dewey 2001 study, heterogeneity decreased significantly (I² = 0%). The relative treatment effect remained consistent with the primary analysis, showing recombined results using a random-effects model (MD = −9.64, 95% CI [−11.53, −7.76], *P* < 0.00001) (Fig. 4).

To investigate whether the effectiveness of apomorphine in treating PD varies by geographic region, we conducted subgroup analysis encompassing clinical studies from three countries: the Netherlands, the USA, and the UK (Table 2). In Dutch studies, apomorphine demonstrated a statistically significant improvement in UPDRS-III scores (MD = −1.21, 95% CI [−2.08, −0.35], *P* = 0.005), with minimal between-study heterogeneity (I² = 0.0%, *P* = 0.995). Similarly, in American studies, apomorphine significantly improved UPDRS-III cores (MD = −0.82, 95% CI [−1.26, −0.38], *P* = 0.0004), with low between-study heterogeneity (I² = 0%, *P* = 0.783). In the UK study, apomorphine also showed improvement in UPDRS-III scores (MD = −2.11, 95% CI [−4.08, −0.14], *P* = 0.035), but there was substantial between-study heterogeneity (I² = 90.8%, *P* = 0.001). These findings suggest that apomorphine is effective across different geographic regions in treating PD. Moreover, variations in administration methods may be a critical factor influencing efficacy and heterogeneity.

**Fig. 5:**
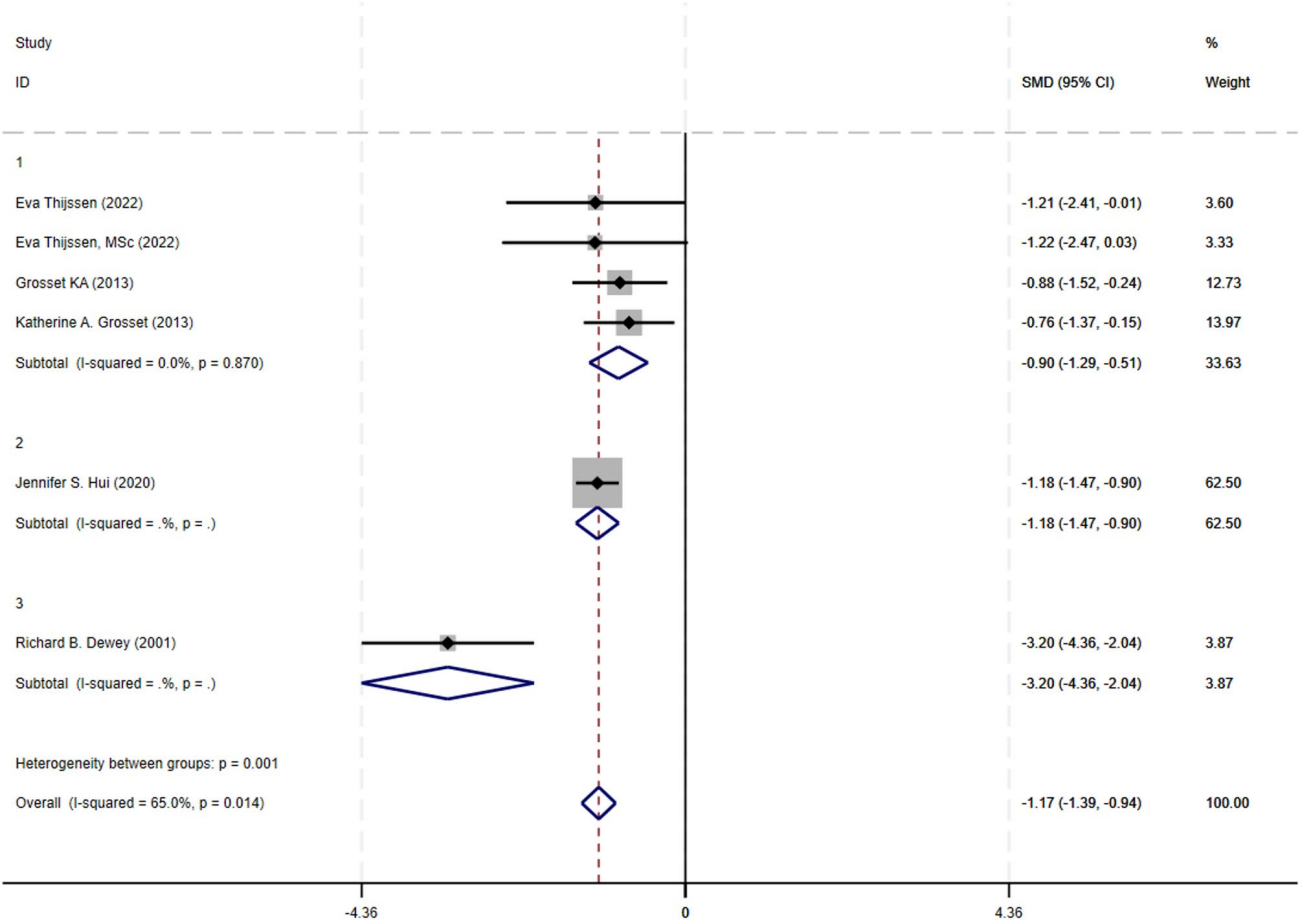
UPDRS-III Scores: apomorphine vs. placebo, by injection route

A subgroup analysis was performed to elucidate the sources of heterogeneity in the overall analysis of UPDRS-III scores, stratifying studies by the route of apomorphine administration (Fig. 5).Inhalation administration: The pooled analysis of four studies utilizing the inhaled route demonstrated a significant, homogeneous improvement in UPDRS-III scores (SMD = −0.90, 95% CI: −1.29 to −0.51; I² = 0.0%).Sublingual administration : A single large study of sublingual apomorphine also showed a significant reduction in UPDRS-III scores (SMD = −1.18, 95% CI: −1.47 to −0.90), contributing the majority of weight (62.50%) to the overall analysis. Subcutaneous injection: In contrast, one study employing subcutaneous injection reported a substantially larger effect size (SMD = −3.20, 95% CI: −4.36 to −2.04), which was a clear outlier.

**Table 2:** Subgroup analysis of reducing UPDRS III scores

Study location	Pooled result of reducing UPDRS III scores		Heterogeneity test		No. of studie
	ES(95%CI)	Effect model and P-value	I^2^-valu	P-value	
Netherlands groups	−1.21(−2.08,−0.35)	Random(*P* = 0.005)	0.0%	*P* = 0.995	2 [21, 22]
American groups	−0.82(−1.26.−0.38)	Random(*P* = 0.0004)	0.0%	*P* = 0.783	2 [34, 35]
United Kingdom groups	−2.11(−4.08,−0.14)	Random(*P* = 0.035)	90.8%	*P* = 0.001	2 [31, 37]

### Reducing off-time after drug administration

**Fig. 6:**
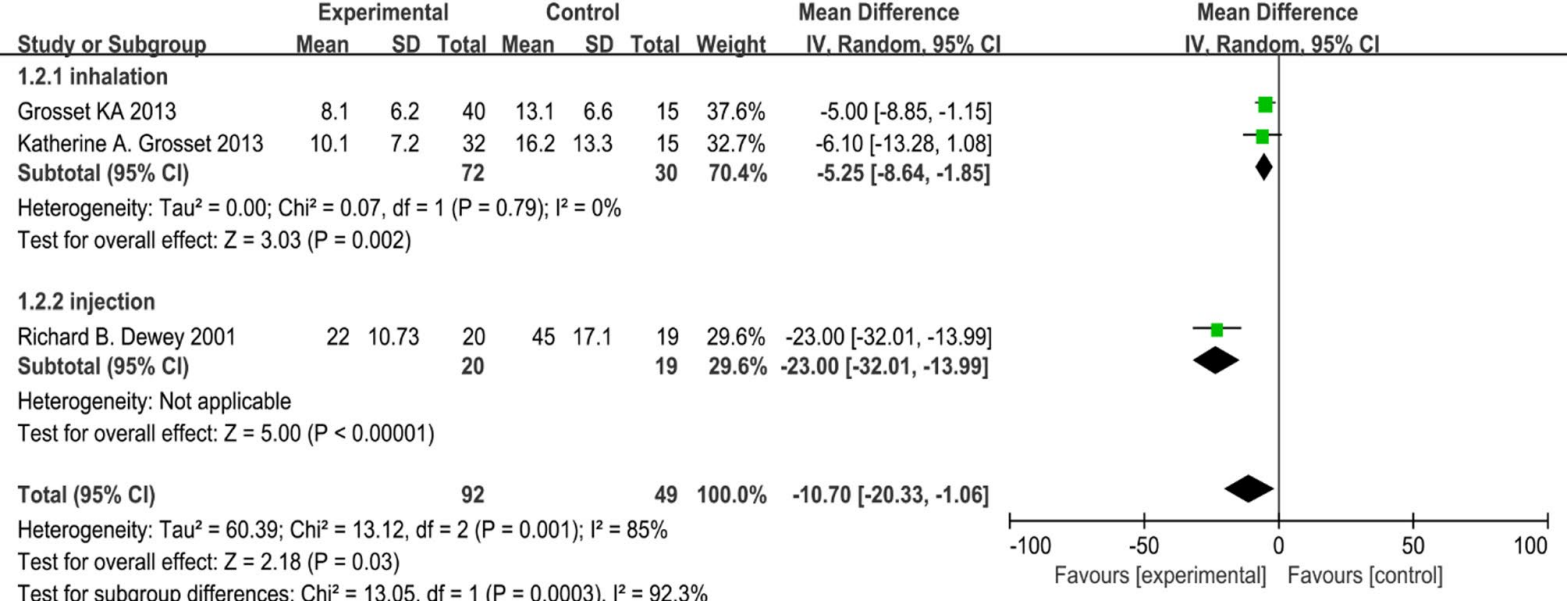
Meta-analysis of reducing off-time in three RCTs

In three double-blind studies, 92 patients received apomorphine treatment, while 49 received a placebo [34, 35, 37]. The meta-analysis showed a statistically significant reduction in off-time in the apomorphine group compared to the placebo group, indicating a notable improvement in motor fluctuation symptoms (MD = −10.70, 95% CI [−20.33, −1.06], *p* = 0.03), albeit with high statistical heterogeneity (I² = 85%) (Fig. 6). Subgroup analysis was conducted based on the route of administration. The inhalation subgroup, which included two studies, Grosset KA 2013 and Katherine (A) Grosset 2013, showed a homogeneous and statistically significant reduction in OFF-time (MD = −5.25 min, 95% CI: −8.64 to −1.85; *p* = 0.002,I² = 0%). In contrast, the injection subgroup, represented by a single study (Richard (B) Dewey 2001), demonstrated a much larger reduction in OFF-time (MD = −23.00 min, 95% CI: −32.01 to −13.99; *p* < 0.00001). The test for subgroup differences was highly significant (Chi² = 13.05, *p* = 0.0003; I² = 92.3%), indicating that the route of administration is a major source of the overall heterogeneity.

### Increase in the duration of post-dose on state maintenance

One study reported changes in open state time during double-blind treatment with apomorphine. According to the article by Grosset et al. 2013, The mean duration of ‘on’ after treatment administration was 74.6 min for apomorphine and 56.7 min for a placebo. The mean difference between the two groups was 17.9 min (95% CI, −32.3 to 68.2 min, *P* = 0.461). There was no evidence of a dose response relating to the dura tion of effect.

The mean duration of ‘on’ after treatment administration was 74.6 min (SD, 39.3; range, 20–169) for inhaled apomorphine and 56.7 min (SD, 23.6; range, 30–75) for placebo, which gave a difference in mean (least squares) of 17.9 (95% CI, 32.3 to 68.2 min, *P* = 0.461). There was no evidence of a dose response relating to the dura tion of effect.

### Rate of conversion from off to on state after drug administration

**Fig. 7:**
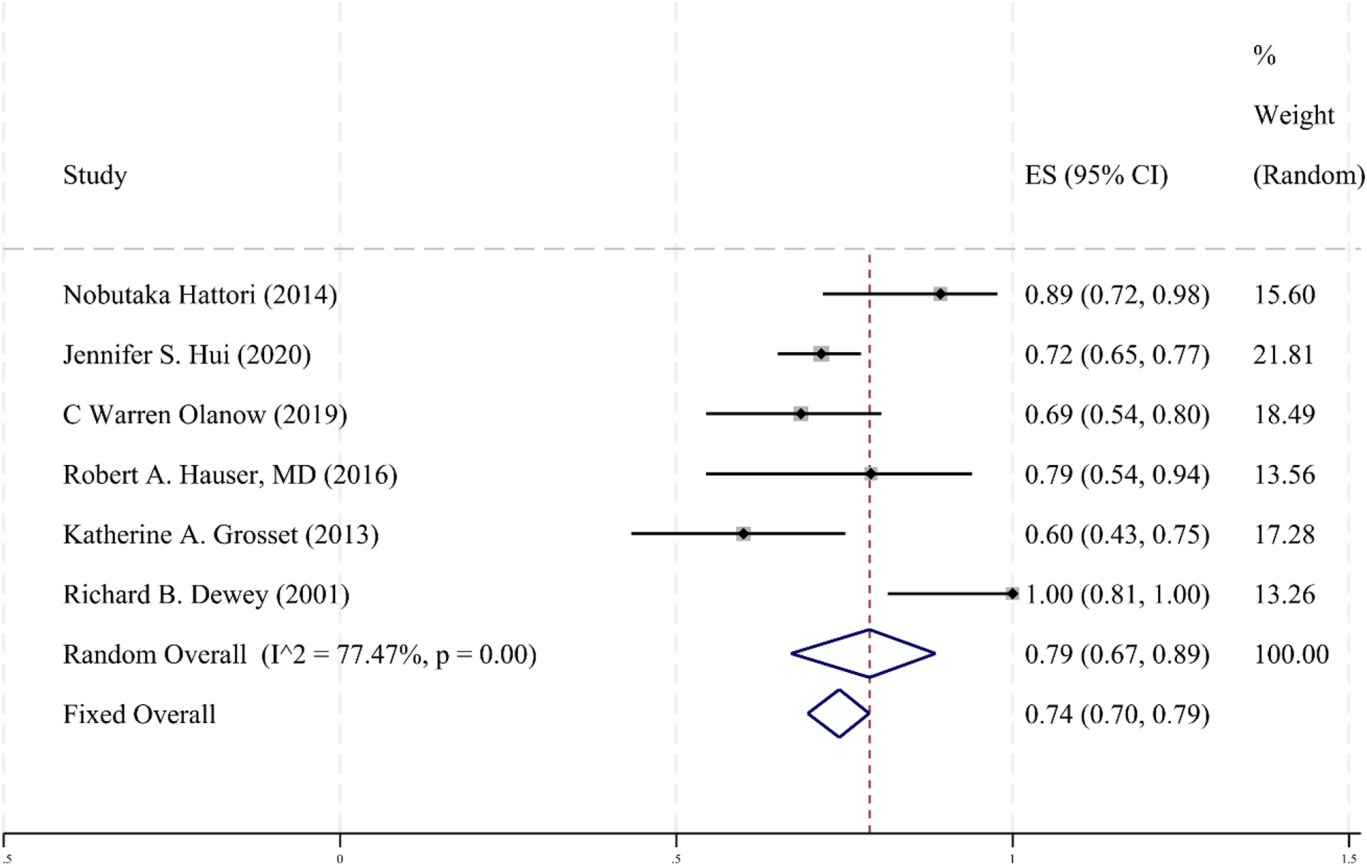
Rate of conversion from off to on state after apomorphine administration

Six clinical studies [28, 31–33, 35, 37] reported the off-to-on conversion rate after taking the drug. These studies were analyzed using a random-effects model, and the combined results (Fig. 7) indicated a conversion rate from off to on state in PD patients treated with apomorphine of 0.79 (95% CI 0.67–0.89) I² = 77.47%. Most studies reported effect size estimates indicating a positive effect, suggesting that apomorphine is effective in facilitating the transition from the off state to the on state in patients with Parkinson’s disease PD. Although there was a slight asymmetry in the publication bias test, it did not reach statistical significance. While the absence of a placebo control limits causal inference, this conversion rate indicates a “high probability of achieving a functional ‘ON’ state when apomorphine is administered in a clinical setting.” This provides practical context for clinicians, acknowledging the utility of this measure while being transparent about its methodological constraints.

**Fig. 8:**
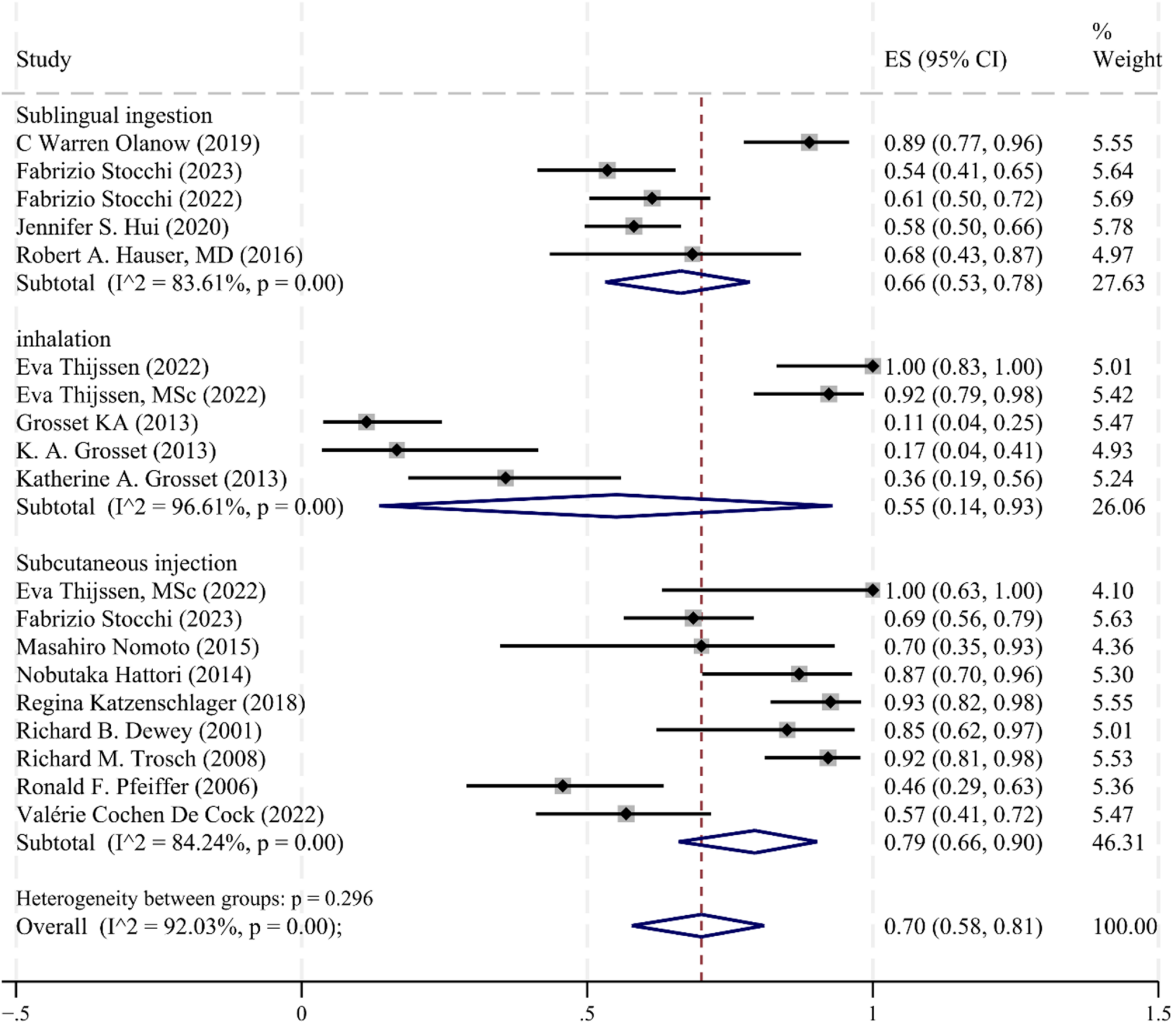
Meta-analysis of adverse events rate

Different modes of administration influenced the incidence of adverse effects when apomorphine was used to treat PD. Although all subgroups experienced adverse reactions associated with apomorphine treatment, some degree of variability in effect size was revealed in the single arm forest plot (Fig. 8), the overall association between apomorphine treatment and the incidence of adverse reactions was 0.70 (95% CI [0.58–0.81], *p* = 0.00). In the risk of publication bias test, there was no significant visual bias, and Egger’s test did not show statistical evidence of publication bias according to the statistical tests provided (*p* = 0.633).

A single-arm subgroup analysis based on different injection modalities for different adverse effects: Nausea: ES (95% CI) = 0.21 (0.16, 0.27), I² = 74.14%, *p* = 0.00; Somnolence: ES (95% CI) = 0.18 (0.13, 0.24), I² = 73.36%, *p* = 0.00;Vomiting: ES (95% CI) = 0.06 (0.02, 0.11), I² = 70.77%, *p* = 0.00; Dizziness: ES (95% CI) = 0.11 (0.07, 0.14), I² = 56.41%, *p* = 0.00;Sweating: ES (95% CI) = 0.06 (0.03, 0.09), I² = 0.00%, *p* = 0.39. Specific subgroup analyses of injection modalities are detailed in Table 3.

**Table 3:** Summary of AEs in the meta-analysis

Adverse effects	pooled rates of AEs (95% CI)	Subgroup analysis of AEs	Pooled rates (95% CI)*	Total patients treated with apomorphine	Total patients with the AEs	No. of studies
disgusting	0.21(0.16,0.27), random	Subcutaneous injection	0.20(0.12, 0.29), random	458	93	11
		Sublingual ingestion	0.21(0.14, 0.29), random	408	92	6
		inhalation	0.38(0.23, 0.55), random	39	15	1
Drowsiness	0.18(0.13,0.24), random	Subcutaneous injection	0.22(0.15, 0.30), random	407	85	10
		Sublingual ingestion	0.14(0.08, 0.20), random	408	55	6
		inhalation	0.17(0.10, 0.27), random	83	17	2
vomit	0.06(0.02, 0.11), random	Subcutaneous injection	0.04(0.00, 0.10), random	78	6	2
		Sublingual ingestion	0.06(0.02, 0.11), random	354	22	4
		inhalation	0.03(0.00, 0.13), random	39	1	1
dizzy	0.11(0.07, 0.14), random	Subcutaneous injection	0.12(0.07, 0.17), random	373	45	9
		Sublingual ingestion	0.1(0.05, 0.18), random	373	38	5
		inhalation	0.10(0.03, 0.19), random	143	15	4
perspire	0.06(0.03, 0.09), fixed	Subcutaneous injection	0.07(0.01, 0.19), random	44	3	1
		Sublingual ingestion	0.06(0.03, 0.11), random	248	16	3

*Abbreviations*: *AEs* Adverse effects, 95% *CI*, 95% Confidence intervals

^*^Different Injection Methods

The descriptive summary of these additional AEs is presented in Table 4. As shown in the figure, neuropsychiatric complications have been reported in studies using inhaled apomorphine, with two reports indicating incidence rates of 16.7% and 30.0%. Movement disorders were documented with subcutaneous infusion (incidence: 4.3% to 16.7%) and sublingual administration (2.5%). Furthermore, cutaneous reactions, including injection site nodules or erythema, were notably associated with subcutaneous injection, with reported incidences of 13.1% and 40.0% in two studies.

**Table 4:** Incidence of neuropsychiatric complications, movement disorders, and cutaneous reactions by apomorphine administration route

	injection modalities	neuropsychiatric complications	Movement disorder	cutaneous reactions
K. A. Grosset (2013)	inhalation	16.7%		
Katherine A. Grosset (2013)	inhalation	30%		
Fabrizio Stocchi (2022)	Sublingual ingestion		2.5%	
Regina Katzenschlager (2020)	Subcutaneous injection		16.7%	13.1%
Valérie Cochen De Cock (2022)	Subcutaneous injection		4.3%	
Masahiro Nomoto (2015)	Subcutaneous injection			40%

## Discussion

This meta-analysis provides a comprehensive assessment of apomorphine’s efficacy and safety in treating dyskinesia among patients with Parkinson’s disease (PD), focusing on its effectiveness in improving motor fluctuations and ‘off’ periods. By systematically reviewing and synthesizing multiple randomized controlled trials (RCTs), our study addresses existing knowledge gaps and offers evidence-based guidance to clinicians for optimizing treatment strategies in PD.

Currently, levodopa is widely used as a first-line therapeutic agent in the clinical management of PD, dramatically improving patients’ motor function and quality of life [4]. However, the long-term use of levodopa raises several challenges as the disease process lengthens and the duration of treatment accumulates, particularly the emergence of complications such as motor fluctuations, which limit its therapeutic efficacy [7, 10]. For example, it has been suggested that approximately 40–60% of patients with advanced PD experience an unpredictable ‘off’ period, during which the efficacy of medications suddenly diminishes, and movement disorders worsen [11]. In addition, studies have shown that long-term levodopa treatment may induce drug-induced dyskinesia, affecting patients’ ability to perform daily activities [13]. In the face of these therapeutic limitations, searching for new strategies to effectively control motor fluctuations while mititablegating side effects has become particularly important.

Against this background, apomorphine, a short-acting dopamine receptor agonist, offers a distinct mechanism from levodopa by directly stimulating D1 and D2 receptors, bypassing the need for enzymatic conversion [14, 39]. This direct action facilitates a rapid onset of effect, underpinning its established clinical utility in rapidly reversing ‘OFF’ episodes and improving motor fluctuations in Parkinson’s disease. The pooled analysis of RCTs indicated that apomorphine treatment was associated with a statistically significant reduction in UPDRS-III scores compared to placebo. However, it is important to note that this overall effect was characterized by substantial heterogeneity, which was largely explained by differences in the route of administration. This efficacy was further evidenced by a high OFF-to-ON state conversion rate of 0.79. The improvements in motor function and the reduction in ‘OFF’ time are meaningful clinical endpoints that have the potential to translate into improvements in patients’ quality of life. However, this meta-analysis did not directly assess quality-of-life outcomes, and such a causal inference cannot be robustly made from our present data. Future studies incorporating patient-reported outcome measures are needed to confirm this potential benefit. Additionally, findings from specific RCTs indicate that dose escalation may yield further motor benefits, supporting its practical use for managing OFF episodes [27]. Subgroup analyses were conducted on UPDRS-III scores to assess improvements based on different modes of apomorphine administration: subcutaneous administration (mean difference = −23.80, 95% CI [−28.31, −19.29]) as reported by Richard B. Dewey in 2001, and inhalation or sublingual administration (mean difference = −9.64, 95% CI [−11.53, −7.76]) in other studies. These results suggest that variations in administration modes were a primary source of heterogeneity in the original analyses. The distinct therapeutic effects observed with subcutaneous injections, compared to other administration modes, may arise from differences in drug absorption rates, bioavailability, and onset of action. This variability in administration methods likely contributed to the differences observed in study outcomes. Fabrizio Stocchireported in a study involving 112 individuals that subcutaneous injections yielded greater improvements compared to sublingual administration [23]. This underscores the impact of administration mode on UPDRS-III scores and suggests that the mode of delivery plays a crucial role in treatment efficacy. Apomorphine demonstrates consistent effectiveness across different geographical regions in treating PD. While improvements in UPDRS-III scores were observed in all three countries studied, studies from the Netherlands and the USA showed high inter-study agreement (I² = 0), indicating consistency in outcomes. In contrast, the UK cohort exhibited significant inter-study heterogeneity (I² = 90.8%), which was mitigated by excluding specific studies. These findings highlight the cross-geographic effectiveness of apomorphine in PD treatment and emphasize the importance of considering different administration modes as critical factors influencing treatment efficacy and study outcomes. Our study has several limitations. Despite conducting a subgroup analysis by administration route, which identified subcutaneous injection as a significant source of heterogeneity, the overall analysis for the UPDRS-III outcome still exhibited considerable residual heterogeneity (I² = 65%). This suggests that other unmeasured factors, such as variations in study population, disease severity, concomitant medications, or specific dosing regimens within each route, may contribute to the variation in treatment effects.

The significant impact of apomorphine in reducing the duration of ‘off’ states in PD patients underscores its unique pharmacological advantages as a dopamine agonist. A combined analysis of three double-blind studies revealed that apomorphine significantly shortened ‘off’ state duration (mean difference = −10.70 min, 95% CI [−20.33, −1.06], *p* = 0.03), indicating a substantial positive effect on improving motor fluctuations. In examining inter-study heterogeneity, we observed that different modes of apomorphine administration—subcutaneous, sublingual, and inhalation—may influence both efficacy and adverse effects. Despite the statistical heterogeneity, the direction of the treatment effect favored apomorphine across different administration routes for the outcomes of UPDRS-III score and OFF-time reduction. This suggests a consistent signal of biological activity, although the magnitude of the clinical benefit is formulation-dependent. The reduction in daily OFF time was statistically significant for the inhaled formulation. However, the mean reduction of approximately 5 min, while statistically robust, is modest and its clinical relevance at the individual patient level may be limited. In contrast, the effect of subcutaneous injection on OFF time was substantially greater, highlighting the importance of selecting the appropriate formulation based on the severity of motor fluctuations.

Apomorphine plays a crucial role in enhancing on-state maintenance time in PD patients. As shown in the 2013 article by Grosset et al. [34], the mean time to onset of action after a single dose was 74.6 min for apomorphine and 56.7 min for placebo. The mean difference between the two groups was 17.9 min. Although apomorphine has been demonstrated to prolong the duration of states in patients with PD, it may also facilitate improvements in their daily activities and overall quality of life by enhancing the management of motor fluctuations. However, this conclusion may be premature, and further multicenter, large-sample studies are necessary to validate and refine it in the future. Furthermore, six randomized controlled trials demonstrated a significant positive effect, with a conversion rate of 0.79 (95% CI: 0.67–0.89). While increasing total ON time is a key therapeutic goal, the quality of this ON time is paramount from a patient’s perspective. Periods of ON time dominated by disabling dyskinesias are not considered a successful therapeutic outcome, as they do not translate to improved functional mobility or quality of life [40]. By focusing on ‘good ON time,’ our analysis provides a more integrated and clinically meaningful measure of apomorphine’s net benefit, effectively capturing its ability to restore functional mobility while minimizing the treatment-limiting impact of dyskinesias [40]. In evaluating the safety profile of apomorphine treatment, we observed an overall adverse reaction rate of 0.79. Although adverse reactions were noted across all subgroups, single-arm forest plots revealed variability in effect sizes among them. Nonetheless, the association between apomorphine treatment and adverse effects remained consistently significant. As lipid-soluble dopamine receptor agonists, substances such as apomorphine are known to be pro-emetic, directly influencing gastrointestinal function or triggering the vomiting reflex by activating the vomiting center in the brainstem [41]. Dopamine agonists can also cause orthostatic hypotension, leading to dizziness in patients [42]. Specific adverse effects such as nausea, drowsiness, vomiting, dizziness, and sweating occurred with various modes of administration, with effect sizes of 0.21 (95% CI: 0.16, 0.27) for nausea, 0.18 (95% CI: 0.13, 0.24) for drowsiness, 0.06 (95% CI: 0.02, 0.11) for vomiting, 0.11 (95% CI: 0.07, 0.14) for dizziness, and 0.06 (95% CI: 0.03, 0.09) for sweating. Overall, these data suggest that while adverse effects are present, they are relatively infrequent and generally mild. Egger’s test was employed to assess the potential risk of publication bias, revealing non-significant p-values >0.05 for both overall and subgroup analyses, except for drowsiness and dizziness, indicating no significant publication bias. Our safety assessment has several important limitations that warrant discussion. We have systematically documented the incidence of neuropsychiatric complications, impulse control disorders (ICDs), and cutaneous reactions, the available data for these specific AEs are often sparse and derived from studies not primarily designed to capture them. Consequently, these event rates are likely underrepresented, and our analysis cannot robustly characterize their full risk profile or identify specific patient risk factors.Intermittent subcutaneous administration of apomorphine is currently utilized in the treatment of Parkinson’s disease [13]. However, it has a high incidence of adverse effects, resulting in the discontinuation of the drug in patients [43].The tolerability profile of apomorphine is formulation-dependent and characterized by a mix of common, often manageable AEs, and less frequent but potentially treatment-limiting or high-burden events. The clinical decision-making process must weigh the substantial motor benefits against this spectrum of potential adverse effects, with a focus on patient education, monitoring, and proactive management. There is a clear opportunity for technological advancements in the administration method aimed at achieving a more patient-friendly approach than the traditional delivery route of apomorphine, ultimately enhancing its safety and tolerability.

While this study offers valuable insights into the role of apomorphine in improving motor fluctuations and dyskinesias in PD patients, several limitations should be noted. Firstly, due to the limited data from some of the included studies, we were unable to perform in-depth analyses on potential influencing factors, such as gender, disease duration, severity, healthcare system context, and specific medication regimens. The generalizability of our findings may be influenced by the modest sample sizes of many included trials and the inter-country heterogeneity observed in our subgroup analysis. While meta-analysis enhances statistical power to derive a robust overall estimate, the small size of individual studies may affect the precision of their specific results. Furthermore, although country-specific differences in clinical practice or patient populations might contribute to heterogeneity, our sensitivity and subgroup analyses confirmed that the core beneficial effect of apomorphine remained consistent across these variations. Future large-scale, multinational studies are encouraged to further validate these findings across diverse healthcare settings. Secondly, since the study designs were primarily cross-sectional, causal relationships between quality of life and these factors cannot be inferred. Lastly, the inclusion criteria were limited to studies published in English and provided a restricted representation of studies from developing countries, which may affect the generalizability of the findings. These limitations underscore the need for caution in interpreting the results and highlight the importance of future studies addressing these shortcomings in order to provide more comprehensive and precise information.

## Conclusion

Apomorphine has demonstrated efficacy in treating PD while maintaining a favorable safety profile. Despite the presence of some adverse effects, their incidence and severity are minimal. However, due to study heterogeneity, further validation from large, multicenter samples is necessary to substantiate these findings and to investigate factors influencing treatment efficacy and safety. This will enhance our understanding and application of apomorphine’s role in PD treatment.

## Data Availability

The original contributions presented in the study are included in the article/Supplementary Material, further inquiries can be directed to the corresponding authors.
